# Epicardial adipose tissue in patients with heart failure

**DOI:** 10.1186/1532-429X-12-40

**Published:** 2010-07-12

**Authors:** Christina Doesch, Dariusch Haghi, Stephan Flüchter, Tim Suselbeck, Stefan O Schoenberg, Henrik Michaely, Martin Borggrefe, Theano Papavassiliu

**Affiliations:** 1 1st Department of Medicine, University Medical Centre Mannheim, Medical Faculty Mannheim, University of Heidelberg, Germany; 2 Department of Clinical Radiology and Nuclear Medicine, University Medical Center Mannheim, Medical Faculty Mannheim, University of Heidelberg, Germany

## Abstract

**Purpose:**

The aim of this study was to evaluate the extent of epicardial adipose tissue (EAT) and its relationship with left ventricular (LV) parameters assessed by cardiovascular magnetic resonance (CMR) in patients with congestive heart failure (CHF) and healthy controls.

**Background:**

EAT is the true visceral fat deposited around the heart which generates various bioactive molecules. Previous studies found that EAT is related to left ventricular mass (LVM) in healthy subjects. Further studies showed a constant EAT to myocardial mass ratio in normal, ischemic and hypertrophied hearts.

**Methods:**

CMR was performed in 66 patients with CHF due to ischemic cardiomyopathy (ICM), or dilated cardiomyopathy (DCM) and 32 healthy controls. Ventricular volumes, dimensions and LV function were assessed. The amount of EAT was determined volumetrically and expressed as mass indexed to body surface area. Additionally, the EAT/LVM and the EAT/left ventricular remodelling index (LVRI) ratios were calculated.

**Results:**

Patients with CHF had less indexed EAT mass than controls (22 ± 5 g/m^2 ^versus 34 ± 4 g/m^2^, p < 0.0001). In the subgroup analysis there were no significant differences in indexed EAT mass between patients with ICM and DCM (21 ± 4 g/m^2 ^versus 23 ± 6 g/m^2^, p = 0.14). Linear regression analysis showed that with increasing LV end-diastolic diameter (LV-EDD) (r = 0.42, p = 0.0004) and LV end-diastolic mass (LV-EDM) (r = 0.59, p < 0.0001), there was a significantly increased amount of EAT in patients with CHF. However, the ratio of EAT mass/LV-EDM was significantly reduced in patients with CHF compared to healthy controls (0.54 ± 0.1 versus 0.21 ± 0.1, p < 0.0001). In CHF patients higher indexed EAT/LVRI-ratios in CHF patients correlated best with a reduced LV-EF (r = 0.49, p < 0.0001).

**Conclusion:**

Patients with CHF revealed significantly reduced amounts of EAT. An increase in LVM is significantly related to an increase in EAT in both patients with CHF and controls. However, different from previous reports the EAT/LVEDM-ratio in patients with CHF was significantly reduced compared to healthy controls. Furthermore, the LV function correlated best with the indexed EAT/LVRI ratio in CHF patients. Metabolic abnormalities and/or anatomic alterations due to disturbed cardiac function and geometry seem to play a key role and are a possible explanation for these findings.

## Background

Human epicardial adipose tissue (EAT) is the true visceral fat of the heart whose regional distribution and function is of growing scientific and clinical interest. EAT covers 80% of the heart's surface and constitutes 20% of total heart weight. It is located in the atrioventricular and interventricular grooves, along the major branches of the coronary arteries, around the atria, over the free wall of the right ventricle and over the apex of the left ventricle [1]. Cardiaovascular magnetic resonance (CMR) using a volumetric approach has proven to be a reproducible and feasible method to assess EAT quantitatively [2].

Anatomically, there is a close relationship between the EAT and the muscular components of the heart. In a recent autopsy study, Corradi et al. [3] investigated the relationship between the EAT and its underlying myocardium in normal hearts and those who were ischemic, hypertrophic or both and found that a constant ratio of fat to muscle exists in each ventricle. The presence of myocardial ischemia and hypertrophy does not alter the ratio of epicardial fat to cardiac muscle. In this context, further echocardiographic findings by Iacobellis et al. also demonstrated that an increase in epicardial fat is significantly related to a consensual and proportional increase in left ventricular mass in healthy subjects [4]. Being a metabolically active organ and a source of several bioactive molecules [1,5,6] EAT may exert protective as well as unfavourable effects on the heart. Growing evidence indicates that EAT might substantially affect cardiovascular morphology and function. An imbalance between the physiologic and pathologic roles of EAT has been suggested in the development of cardiac pathologies. However, little is known about the changes of EAT coming along with the development of heart failure and the concomitant deterioration of left ventricular function.

Thus, the aim of this study was to assess the extent of EAT and its relationship to the LV ejection fraction (EF%), end-diastolic diameter (LV-EDD) and LV end-diastolic mass (LV-EDM) assessed by cardiovascular magnetic resonance (CMR) in patients with congestive heart failure (CHF) due to ischemic or dilated cardiomyopathy and healthy controls.

## Methods

### Study population

A total of 66 patients (54 males and 12 females; mean age, 63± 2 years) consecutive patients with a history of compensated symptomatic heart failure (NYHA functional class II or greater) and the presence of cardiac dysfunction on echocardiography study (left ventricular function ≤ 35%) for more than one year were included in the study. For the traditional binary classification (ischemic vs. nonischemic), an ischemic aetiology of heart failure was defined as the presence of any epicardial coronary vessels with ≥75% stenosis or any history of myocardial infarction or coronary revascularization. The number-of-diseased-vessels classification was defined as the number of vessels with ≥75% stenosis (0, 1, 2 or 3). The diagnosis of dilated cardiomyopathy was based on M-mode and 2D/Doppler echocardiographic examination demonstrating an end-diastolic diameter >56 mm and a normal coronary angiography performed within the previous 6 months.

The 32 healthy volunteers (25 males and 7 females, 57 ± 11 years) satisfied the following criteria: normal physical examination, normal blood pressure (< 120 mm Hg and <80 mm Hg), normal ECG findings, no history of chest pain or dyspnea, no diabetes, and normal 2D echocardiographic and Doppler examination. None of the subjects was on medication. Any potential subjects with evidence of heart disease, hypertension or other systemic disorders were excluded from the study.

### Image Acquisition

All studies were performed using a 1.5 Tesla whole-body imaging system (Magnetom Sonata, Siemens Medical Systems, Erlangen, Germany). A dedicated 4-element, phased-array cardiac coil was used. Images were acquired during repeated end-expiratory breath-holds. Scout images (coronal, sagittal, and axial planes) were obtained for planning of the final double-oblique long-axis and short-axis views. To evaluate functional parameters, electrocardiogram-gated cine images were then acquired using a segmented steady-state free precession [fast imaging with steady-state precession (true-FISP)] sequence (time to echo/time of repetition 1.6/3.2 ms, temporal resolution 35 ms, in-plane spatial resolution 1.4 1.8 mm, slice thickness 5 mm, interslice gap 5 mm). Seven to 12 short-axis views covering the whole left and right ventricle were obtained. For the assessment of the epicardial adipose tissue, we used a dark blood prepared T1-weighted multislice turbo spin-echo pulse sequence with a water suppression prepulse to obtain a transversal 4-chamber view and short-axis images in the same orientations used for the cine short-axis images. Imaging parameters were as follows: time of repetition = 800 ms, time to echo = 24 ms, slice thickness = 4 mm, interslice gap = 2 mm, and field of view = 30 to 34 cm.

Ten minutes after injection of a gadolinium-based contrast agent (Magnevist, Bayer-Schering Pharma AG, Berlin, Germany), a segmented inversion recovery cine trueFISP pulse sequence (TI scout) acquisition was performed at a midventricular short-axis location. This acquisition was used to determine the TI at which the signal of normal myocardium is null for the subsequent late gadolinium enhancement (LGE) acquisition. After that, LGE images were acquired in the same orientation as the cine images using a 2D-segmented inversion recovery gradient-echo pulse sequence triggered to end-diastole (repetition time/echo time = 9.6/4.4 ms, flip angle 25°, matrix 208 × 256 and a typical voxel size of 1.6 × 1.3 × 5.0). LGE was only considered to be present if it was also present in the same slice after swapping phase encoding, thus excluding artifacts.

### Image Analysis and Determination of Ventricular Parameters

Image analysis and quantitative analysis was performed off-line using dedicated software (ARGUS, Siemens). Each study was examined for abnormalities in the morphology of the right and left ventricle. End-diastolic and end-systolic volumes and left ventricular mass was analyzed with the serial short-axis true-FISP cine loops, using manual segmentation. Stroke volumes and ejection fractions were calculated. Additionally, left- and right-ventricular diameters were measured.

### Volumetric Assessment of the Absolute Mass of EAT

The amount of EAT was calculated by using the modified Simpson's rule with integration over the image slices. The contours of EAT were outlined at end diastole in the short-axis views covering the entire left and right ventricle. For EAT mass determination, the area subtended by the manual tracings was determined in each slice and multiplied by the slice thickness to yield the fat volume. Total EAT volume was obtained after the data summation of all slices. To obtain EAT mass, the volume of EAT was multiplied by the specific weight of fat (0.92 g/cm^3^). The observer was blinded to patient details.

Additionally, the left ventricular remodelling index (LVRI) was calculated as the ratio of left ventricular mass (LV-EDM) to left ventricular volume (LV-EDV) as previously published by De Castro S et al. [7] who established a LVRI of 1.03 ± 0.12 g/ml as normal value in 152 healthy volunteers.

To assess inter- and intraobserver reproducibility of the volumetric EAT measurements, two blinded investigators evaluated 40 randomly selected subjects among the study population and one of them repeated the measurements two weeks after the first evaluation.

### Late Gadolinium Enhancement

LGE was assessed visually, and the LGE mass was measured by manual planimetry on all short-axis slices by an observer blinded to all patient details. Summing the LGE mass of all slices yielded the total mass of LGE. The extent of LGE was then expressed as a percentage of the total LV mass.

### Statistical Analysis

The data are presented as mean value SD. Body mass index (BMI) was calculated by the common formula: BMI (kg/m^2^) = weight (kg)/height (m)^2^. Body surface area (BSA) was assessed by a variation of the DuBois and DuBois formula: BSA (m^2^) = [weight (kg)^0.425 ^× height (cm)^0.725^] × 0.007184 [8]. An unpaired, 2-tailed student's t-test was used for parametric data. The Mann-Whitney U test, [chi]^2 ^test was applied for nonparametric data. A *p*-value <0.05 was considered significant for all comparisons. Linear regression analysis was performed on anthropometric and CMR parameters to identify correlates of EAT. Multivariate analysis was performed with logistic regression analysis using block entry of the following variables: LV-EF (%), RV-EF(%), LV-EDD (mm) and indexed LV-EDM (g/m^2^) to evaluate if these variables were independent predictors of indexed EAT mass. Besides, univariate linear regression analysis was performed between LV-EF and indexed EAT in CHF patients and healthy controls separately. Furthermore, univariate linear regression analysis was applied to determine the correlation between LV-EF and left ventricular remodelling index (LVRI) calculated as the ratio of left ventricular mass (LV-EDM) to left ventricular volume (LV-EDV) as well as the indexed EAT/LVRI-ratio in patients with CHF. Additionally, the LV-EDM and the indexed EAT was compared in CHF patients with and without hypertensive heart disease. Analysis was performed using SPSS statistical software (version 14.0, SPSS Inc., Chicago, Illinois).

Power calculation (two-sided paired t-test performed with SAS, version 8.2 software [SAS Institute, Cary, NC] to assess the sample size needed in this study was done on the basis of the estimated standard deviation (SD) between the indexed EAT of healthy controls and CHF patients derived from the results of our previous study [2]. According to this calculation, the number of subjects needed to establish a power of 90% (with α = 0.05) was 22 CHF patients and 11 healthy controls.

The inter- and intraobserver reproducibility was assessed by Bland-Altman analysis, providing the mean difference (d) and its limits of agreement (d ± 1.96 × SD, where SD = standard deviation of the differences).

## Results

### Demographics and Baseline characteristics

The baseline characteristics of all subjects studied are shown in Table 1. There were no significant differences regarding age, BMI and BSA between patients with heart failure and controls.

**Table 1:** Demographics and baseline characteristics: Healthy Controls and Patients with CHF.

	Healthy Controls n = 32	Patients with CHF n = 66	p-value
Age	57 ± 11	63 ± 12	0.06
Male Sex	25/32 (78%)	54/66 (82%)	0.44
Body weight (kg)	83 ± 14	80 ± 16	0.35
BSA (m^2^)	1.7 ± 0.1	1.9 ± 0.2	0.27
BMI (kg/m^2^)	28 ± 4	27 ± 4	0.49

Data are presented as the mean value ± SD.

BMI: body mass index, BSA: body surface area, CHF: chronic heart failure

Comparing the baseline characteristics of patients with ICM and DCM (Table 2) it becomes evident that due to the different disease course patients with ICM were older and had a reduced body weight and BMI compared to patients with DCM.

**Table 2:** Demographics and baseline characteristics: Subgroup Analysis according to aetiology of CHF.

	ICM n = 36	DCM n = 30	p-value
Age	66 ± 9	58 ± 14	0.01
Male Sex	31/36 (86%)	23/30 (77%)	0.36
Body weight (kg)	76 ± 13	85 ± 18	0.02
BSA (m^2^)	1.9 ± 0.2	2.0 ± 0.3	0.06
BMI (kg/m^2^)	26 ± 4	28 ± 5	0.02
NYHA functional class			
I	0	0	-
II	2/36	5/30	0.99
III/IVI	34/36	25/30	0.56

Data are presented as the mean value ± SD. BSA: body surface area, BMI: body mass index, CHF: chronic heart failure, DCM: dilated cardiomyopathy, ICM: ischemic cardiomyopathy, NYHA: New York Heart Association

In our study cohort 21/66 (31.8%) patients suffered from diabetes mellitus and were treated with oral antidiabetic medication 15/66 (22.7%) or insulin 6/66 (9.1%). 17/66 (36%) suffered from hypertensive heart disease. Hyperlipidemia was diagnosed in 38/66 (57.6%). 24/66 (36.4%) were smoker and 15/66 (22.7%) had a family history of CAD.

Among the patients with ICM 34/36 (94%) suffered an acute myocardial infarction 11.6 ± 7.3 years ago. None of the DCM patients had a history of prior myocardial infarction.

The severity of symptoms at admission was assessed by NYHA classification. None of the patients were in NYHA class I, 7/66 (11%) patients in II, and 59/66 (89%) patients in III-IV.

### CMR parameters

The CMR characteristics of our study cohort are summarized in Table 3. The mean extent of LGE in our patients with ICM was 32.6 ± 10.4%.

**Table 3:** CMR Characteristics: Healthy Controls and Patients with chronic heart failure.

	Controls n = 32	Patients with CHF n = 66	p-value
LV-EF (%)	58 ± 5	27 ± 9	< 0.0001
LV-EDM (g)	125 ± 35	207 ± 56	< 0.0001
Indexed LV-EDM (g/m^2^)	64 ± 15	109 ± 28	< 0.0001
LV-ESV (ml)	75 ± 85	211 ± 76	< 0.0001
LV-EDV (ml)	141 ± 36	294 ± 86	< 0.0001
LV-EDD (mm)	50 ± 5	69 ± 8	< 0.0001
LVRI g/ml	0.9 ± 0.2	0.7 ± 0.2	0.001
RV-EF (%)	58 ± 6	42 ± 14	< 0.0001
RV-ESV (ml)	55 ± 16	103 ± 64	0.0001
RV-EDV (ml)	137 ± 32	168 ± 68	0.02
EAT volume (ml)	71 ± 13	46 ± 11	< 0.0001
Indexed EAT volume (ml/m^2^)	36 ± 5	24 ± 5	< 0.0001
EAT mass (g)	67 ± 13	43 ± 11	< 0.0001
Indexed EAT mass (g/m^2^)	34 ± 4	22 ± 5	< 0.0001
EAT mass/LV-EDM ratio	0.54 ± 0.1	0.21 ± 0.1	< 0.0001

Data are presented as the mean value ± SD.

CMR: cardiovascular magnetic resonance, EAT: epicardial adipose tissue, LV-EDD: left ventricular end-diastolic diameter, LV-EDM: left ventricular end-diastolic mass, LV-EDV: left ventricular end-diastolic volume, LV-EF: left ventricular ejection fraction, LV-ESV: left ventricular end-systolic volume, RV-EDV: right ventricular end-diastolic volume, RV-EF: right ventricular ejection fraction, RV-ESV: right ventricular end-systolic volume

**Figure 1:**
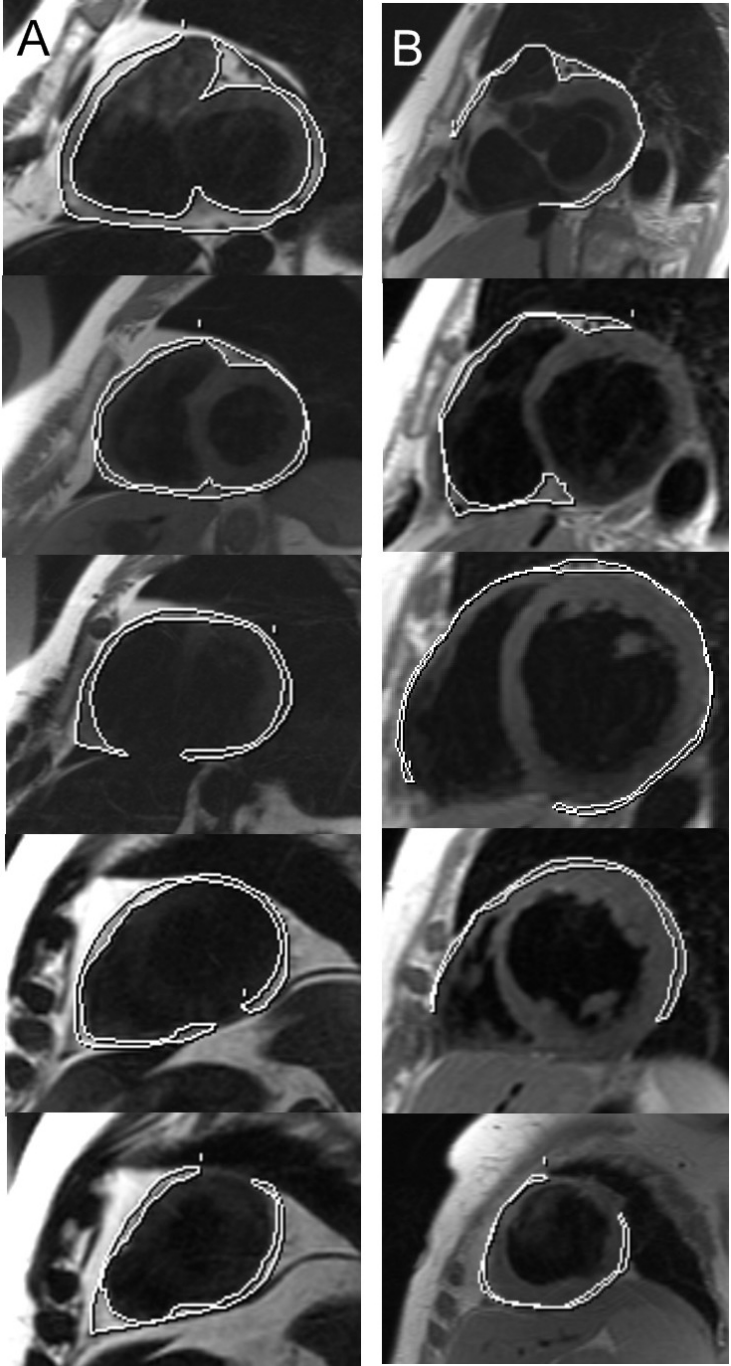
**Difference in EAT mass in healthy controls and patients with CHF**. Volumetric measurement of EAT outlining the contours of EAT in end-diastolic images of short axis covering the left and right ventricle in a healthy control with normal EAT mass (**Panel A) **and in a CHF patient with reduced EAT mass **(Panel B)**. CHF: chronic heart failure, EAT: Epicardial adipose tissue, LV-EDM: left ventricular enddiastolic mass

In patients with CHF, left ventricular ejection fraction (LV-EF) and right ventricular ejection fraction (RV-EF) were significantly reduced whereas left ventricular end-diastolic diameter (LV-EDD) and right ventricular end-diastolic diameter (RV-EDD) as well as indexed left ventricular end-diastolic mass (LV-EDM) were significantly elevated compared to controls. Indexed EAT mass in patients with heart failure was significantly lower compared to controls as illustrated in Figure 1. Subgroup analyis of CMR parameters in patients with ICM and DCM (Table 4) revealed a comparably diminished LV-EF and RV-EF. LV-EDD and RV-EDD were equally increased and indexed LV-EDM as well as indexed EAT mass were also comparable among patients with ICM and DCM.

**Table 4:** CMR Characteristics: Subgroup Analysis according to aetiology of heart failure.

	ICM n = 36	DCM n = 30	p-value
LV-EF (%)	27 ± 9	26 ± 8	0.75
LV-EDM (g)	194 ± 45	223 ± 64	0.03
Indexed LV-EDM (g/m^2^)	104 ± 23	115 ± 32	0.12
LV-ESV (ml)	211 ± 76	227 ± 89	0.42
LV-EDV (ml)	283 ± 73	308 ± 99	0.25
LV-EDD (mm)	68 ± 7	70 ± 9	0.32
RV-EF (%)	44 ± 15	40 ± 13	0.28
RV-ESV (ml)	92 ± 46	117 ± 79	0.10
RV-EDV (ml)	155 ± 47	183 ± 55	0.10
EAT volume (ml)	43 ± 10	49 ± 13	0.03
Indexed EAT volume (ml/m^2^)	23 ± 5	25 ± 6	0.14
EAT mass (g)	40 ± 9	46 ± 12	0.03
Indexed EAT mass (g/m^2^)	21 ± 4	23 ± 6	0.14
EAT mass/LV-EDM ratio	0.21 ± 0.1	0.21 ± 0.1	0.58

CMR: cardiac magnetic resonance, EAT: epicardial adipose tissue, LV-EF: left ventricular ejection fraction, LV-EDD: left ventricular end-diastolic diameter, LV-EDM: left ventricular end-diastolic mass, LV-EDV: left ventricular end-diastolic volume, LV-ESV: left ventricular end-systolic volume, RV-EDV: right ventricular end-diastolic volume, RV-EF: right ventricular ejection fraction, RV-ESV: right ventricular end-systolic volume

The intra- and interobserver correlation of EAT determination was good. The corresponding Bland-Altmann plots for the intraobserver and interobserver variability showed a mean value of 0.69% vs 3.18%, a SD of ± 0.96% vs ± 3.13% and limits of agreement from -1.1% to +2.5% vs -3.0% to +9.3% (intraobserver vs interobserver variability respectively).

### Correlation of indexed EAT mass with anthropometric and CMR parameters

In patients with CHF simple linear regression analysis revealed a significant correlation of indexed EAT mass with LV-EF (r = 0.32, p = 0.008), LV-EDD (r = 0.42, p = 0.0004), indexed LV-EDM (r = 0.59, p < 0.0001) and RV-EF (r = 0.32, p = 0.009). This correlation was also existent in subgroup analysis in patients with DCM and ICM. In patients with ICM there was no significant correlation between indexed EAT and the LGE extent (%) (r = 0.11, p = 0.52). No correlation was found between indexed EAT mass and RV-EDD (r = 0.07, p = 0.6) as well as the anthropometric parameters age (r = 0.04, p = 0.76) and BMI (r = 0.06, p = 0.63). In healthy controls indexed EAT mass only correlated with indexed LV-EDM (r = 0.36, p = 0.04). Multivariate logistic regression analysis, a model using the LV-EF, LV-EDD, indexed LV-EDM and RV-EF to predict the extent of EAT in patients with CHF, showed that indexed LV-EDM was the only parameter that was independently associated with the indexed EAT mass (p = 0.0001).

### EAT mass/LV-EDM ratio

**Figure 2:**
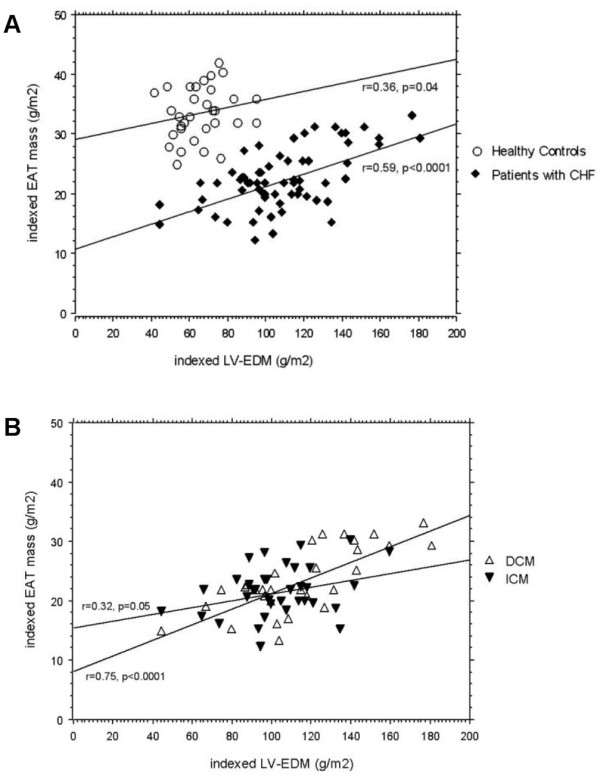
**Regression Plot of indexed EAT mass vs indexed LV-EDM**. **Panel A **illustrates the significant correlation of indexed EAT mass and indexed LV-EDM in patients with CHF and in healthy controls. **Panel B **shows the subgroup analysis of the relationship between indexed EAT mass and indexed LV-EDM in patients with DCM and patients with ICM. CHF: chronic heart failure, DCM: dilated cardiomyopathy, EAT: Epicardial adipose tissue, ICM: ischemic cardiomyopathy, LV-EDM: left ventricular end-diastolic mass.

As illustrated on Figure 2A there is a parallel and correlated increase in indexed EAT mass and indexed LV-EDM in patients with CHF as well as in healthy controls. However, if compared to patients with CHF, healthy controls showed a consistently higher EAT mass/LV-EDM ratio (0.54 ± 0.1 versus 0.21 ± 0.1, p < 0.0001) that was also illustrated by a higher intercept of the regression curve. Among patients with DCM and ICM the EAT mass/LV-EDM ratio (0.21 ± 0.1 versus 0.21 ± 0.1, p = 0.58) was similar (Figure 2B). Comparing the LV-EDM (222.9 ± 86.1 g versus 201.9 ± 40.0, p = 0.18) and indexed EAT (22.4 ± 5.3 g/m^2 ^versus 22.2 ± 4.8 g/m^2^, p = 0.87) results between CHF patients with and without hypertensive heart disease there was no significant difference.

### Correlation between LV-EF and LGE, indexed EAT as well as parameters of left ventricular remodelling

**Figure 3:**
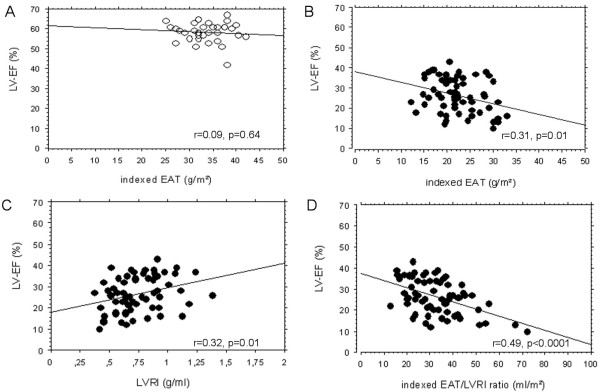
**Correlation between LV-EF and indexed EAT, LVRI as well as the indexed EAT/LVRI ratio**. **Panel A **shows the correlation between LV-EF and indexed EAT in healthy controls. **Panel B **illustrates the correlation between LV-EF and indexed EAT in CHF patients. **Panel C **displays the correlation between LV-EF and LVRI in CHF patients. The correlation between LV-EF and the indexed EAT/LVRI ratio is presented on **Panel D**. CHF: chronic heart failure, EAT: epicardial adipose tissue, LV-EF: left ventricular function, LVRI: left ventricular remodelling index

In CHF patients a reduced LV-EF correlated inversely with the LGE extent % (r = 0.41, p = 0.01). Looking at the correlation between LV-EF and indexed EAT as well as parameters of left ventricular remodelling, there was no significant correlation between LV-EF and indexed EAT in healthy controls (r = 0.09, p = 0.64, Figure 3A). Whereas in CHF patients, we found an inversed correlation between LV-EF and indexed EAT (r = 0.31, p = 0.01, Figure 3B). In contrast to healthy controls who exhibited a normal LVRI, CHF patients had a significantly reduced LVRI (0.9 ± 0.2 g/ml vs 0.7 ± 0.2 g/ml, p = 0.001). In CHF patients, the reduced LV-EF was positively correlated with a decreased LVRI (r = 0.32, p = 0.01, Figure 3C). Irrespective of the aetiology of heart failure, higher indexed EAT/LVRI-ratios in CHF patients correlated best with a reduced LV-EF (r = 0.49, p < 0.0001, Figure 3D).

## Discussion

Our study demonstrates that in patients with CHF and severely reduced impaired LV-EF (LV-EF < 35%), EAT is significantly reduced compared to healthy controls. The reduction of EAT is irrespective of the underlying aetiology of the cardiomyopathy. Like in healthy controls an increase in left ventricular mass also leads to an augmentation of the EAT mass in patients with CHF, however, the EAT mass/LV-EDM ratio is significantly lower compared to healthy controls.

The amount of EAT has been assessed by numerous imaging modalities [2,4,9,10]. Fluechter et al. [2] were the first to describe a volumetric measurement of epicardial fat volume using CMR. This method turned out to be less influenced by the individual cardiac anatomy and fat distribution compared to echocardiographic measurements at single points and was used for the assessment of EAT in our study.

So far, the role of EAT and its contribution to the development of cardiac pathology is quite ambiguous. There is growing evidence of a close functional and anatomical relationship between the adipose tissue and muscular components of the heart. Its close proximity to the myocardium suggests that EAT as a metabolically active organ and a source of several bioactive molecules may influence cardiac morphology and function [1,11,12]. Under physiological conditions EAT is supposed to act as a buffering system between the myocardium and the local vascular bed [13,14]. Increased EAT could serve as a scavenger of excess fatty free acids which interfere with the generation and propagation of the contractile circle of the heart, may cause ventricular arrhythmias and are supposed to alter cardiac repolarization [15,16]. On the other hand, EAT possesses a high lipolytic activity so that it is able to serve as a ready source of free fatty acids under conditions of an increased myocardial energy demand [1].

Our results showed that patients with CHF had less indexed EAT mass than controls. In the subgroup analysis there were no significant differences in indexed EAT mass between patients with ICM and DCM. In healthy controls there was no significant correlation between indexed EAT and LV-EF. In contrast, CHF patients revealed a significant inversed correlation between indexed EAT and LV-EF as well as RV-EF and a linear correlation between indexed EAT and LV-EDD. However, indexed EAT did not correlate with RV-EDD, which was due to the predominantly left ventricular heart failure. Compared to healthy controls who possessed a normal left ventricular remodelling index (LVRI), CHF patients had a significantly reduced LVRI. In CHF patients, the reduced LV-EF showed a positive correlation with the decreased LVRI. As indicated by a significant interaction between indexed EAT and left ventricular remodelling, higher indexed EAT/LVRI-ratios in CHF patients correlated best with a reduced LV-EF. This correlation existed regardless of the underlying aetiology of heart failure. The reversal of the correlation between LV-EF and indexed EAT seen in our CHF patients can be explained by the modification in left ventricular shape and size coming along with left ventricular remodelling due to progressive heart failure. Since changes in left ventricular geometry are dynamic, affecting both mass and volume, the LVRI is significantly reduced in CHF patients. With decreasing LV-EF, CHF patients showed an decrease in LVRI and indexed EAT increase resulting in an increasing indexed-EAT-LVRI ratio, indicating a remodelling not compensated by an adequate EAT increase.

The extent of LGE in our 36 ICM patients was 32.6 ± 10.4%. As anticipated there was a significant correlation between the LV-EF and the LGE extent, whereas no significant correlation existed between indexed EAT and the LGE extent. However, due to the small number of patients in the subgroup analysis, our study might be underpowered for this analysis.

Previous studies [3,17] showed a close relationship between ventricular muscle mass and EAT. This hypothesis of a tight relationship between muscle and fat mass is also corroborated by anatomical observations [18]. Corradi D et al. [3] described a constant fat-muscle ratio in each ventricle which is not influenced by ischemia or hypertrophy. Our study also observed a constant and parallel increase of left ventricular mass and EAT in patients with severe heart failure. However, the fat-muscle ratio is significantly lower in patients with severe heart failure compared to healthy controls with preserved left ventricular function. These results are in line with a post-mortem study by Schejbal V. et al. [18] who also found significantly less EAT in patients with signs of congestive heart failure. Additionally, in the study by Corradi et al. [3] patients with hypertrophy had a left ventricular mass of 226 ± 67 g and a EAT of 73 ± 23 g, the ischemic hypertrophic patients displayed a left ventricular mass of 225 ± 59 g and a EAT of 76 ± 32 g. Our CHF patients had a comparable left ventricular mass 207 ± 56 g however, the EAT was significantly reduced 43 ± 11 g. Therefore, we assume that the reduced ratio is not caused by excessive LV hypertrophy but is the effect of an insufficient EAT increase in the failing heart. Comparing the LV-EDM and EAT results between CHF patients with and without hypertensive heart disease there was no significant difference.

In patients with CHF and a severely reduced LV-EF, the reduced EATmass/left ventricular mass ratio may indicate a decreased buffering capacity for excess fatty free acids as well as a diminished responsiveness to adjust to special energy demands of the heart. A reduction of EAT may result in a reduced adiponectin production that has shown to exert protective effects under ischemic conditions [19].

### Limitations

One limitation of our study is the relatively small number of study participants. Larger randomized trials including patients with various degrees of left ventricular function impairment are needed to generalize our observations and to prove whether they are linearly related to LV-EF decrease.

Due to the different disease course and time of manifestation patients with dilated cardiomyopathy were younger than those with ischemic cardiomyopathy. However, as the thickness of EAT does not change between the age of 40 and death [18] this fact should not alter the results of our study.

We found a correlation between EAT and several MRI parameters indicating left ventricular remodeling. However, as correlation is a necessary but not a sufficient condition to make causal inference with reasonable confidence, our study was not targeted on causal relationships among these parameters and EAT and no conclusion on the causality of the parameters can be drawn. It will be the challenge of further studies to evaluate the potential causal explanations of the observed correlations.

## Conclusion

Patients with CHF revealed significantly reduced amounts of EAT compared to healthy controls irrespective of the underlying aetiology of the cardiomyopathy. An increase in LVM is significantly related to an increase in EAT in both patients with CHF and controls. However, different from previous reports the EAT/LVEDM-ratio in patients with CHF was significantly reduced compared to healthy controls. Metabolic abnormalities and/or anatomic alterations due to disturbed cardiac function and geometry seem to play a key role and are a possible explanation for these findings. Furthermore, as indicated by a significant interaction between indexed EAT and left ventricular remodelling, higher indexed EAT/LVRI-ratios in CHF patients correlated best with a reduced LV-EF.

## Abbreviations

CHF: congestive heart failure; CMR: cardiovascular magnetic resonance; DCM: dilated cardiomyopathy; EAT: epicardial adipose tissue; ICM: ischemic cardiomyopathy; LA: left atrium; LGE: late gadolinium enhancement; LV: left ventricle.

## Competing interests

The authors declare that they have no competing interests.

## Authors' contributions

CD evaluated CMR images, participated in study-design, performed statistical analysis and drafted the manuscript, figures and tables. DH contributed equally to the writing of this manuscript. SF participated in study-design and evaluation of CMR data. TS participated in study-design and the revision of the manuscript. SS participated in study-design and evaluation of CMR-images. HM participated in the revision of the manuscript and repeated the EAT volumetry to assess interobserver measurement reproducibility. MB participated in study-design, scientific and clinical advice concerning CHF and was responsible for the revision of the manuscript. TP participated in study-design and its coordination, performed and evaluated CMR images and was responsible for the revision of the manuscript. All authors read and approved the final manuscript.

## References

[B1] Iacobellis G Corradi D Sharma AM Epicardial adipose tissue: anatomic, biomolecular and clinical relationships with the heart Nat Clin Pract Cardiovasc Med 2005 2 536 543 10.1038/ncpcardio0319 16186852

[B2] Fluchter S Haghi D Dinter D Heberlein W Kuhl HP Neff W Sueselbeck T Borggrefe M Papavassiliu T Volumetric assessment of epicardial adipose tissue with cardiovascular magnetic resonance imaging Obesity (Silver Spring) 2007 15 870 878 10.1038/oby.2007.591 17426322

[B3] Corradi D Maestri R Callegari S Pastori P Goldoni M Luong TV Bordi C The ventricular epicardial fat is related to the myocardial mass in normal, ischemic and hypertrophic hearts Cardiovasc Pathol 2004 13 313 316 10.1016/j.carpath.2004.08.005 15556777

[B4] Iacobellis G Ribaudo MC Assael F Vecci E Tiberti C Zappaterreno A Di Mario U Leonetti F Echocardiographic epicardial adipose tissue is related to anthropometric and clinical parameters of metabolic syndrome: a new indicator of cardiovascular risk J Clin Endocrinol Metab 2003 88 5163 5168 10.1210/jc.2003-030698 14602744

[B5] Fantuzzi G Mazzone T Adipose tissue and atherosclerosis: exploring the connection Arterioscler Thromb Vasc Biol 2007 27 996 1003 10.1161/ATVBAHA.106.131755 17303782

[B6] Kankaanpaa M Lehto HR Parkka JP Komu M Viljanen A Ferrannini E Knuuti J Nuutila P Parkkola R Iozzo P Myocardial triglyceride content and epicardial fat mass in human obesity: relationship to left ventricular function and serum free fatty acid levels J Clin Endocrinol Metab 2006 91 4689 4695 10.1210/jc.2006-0584 16926257

[B7] De Castro S Caselli S Maron M Pelliccia A Cavarretta E Maddukuri P Cartoni D Di Angelantonio E Kuvin JT Patel AR Pandian NG Left ventricular remodelling index (LVRI) in various pathophysiological conditions: a real-time three-dimensional echocardiographic study Heart 2007 93 205 209 10.1136/hrt.2006.093997 16914482 PMC1861397

[B8] Bland JM Altman DG Statistical methods for assessing agreement between two methods of clinical measurement Lancet 1986 1 307 310 2868172

[B9] Iacobellis G Assael F Ribaudo MC Zappaterreno A Alessi G Di Mario U Leonetti F Epicardial fat from echocardiography: a new method for visceral adipose tissue prediction Obes Res 2003 11 304 310 10.1038/oby.2003.45 12582228

[B10] Taguchi R Takasu J Itani Y Yamamoto R Yokoyama K Watanabe S Masuda Y Pericardial fat accumulation in men as a risk factor for coronary artery disease Atherosclerosis 2001 157 203 209 10.1016/S0021-9150(00)00709-7 11427222

[B11] Iacobellis G Pistilli D Gucciardo M Leonetti F Miraldi F Brancaccio G Gallo P di Gioia CR Adiponectin expression in human epicardial adipose tissue in vivo is lower in patients with coronary artery disease Cytokine 2005 29 251 255 15749025 10.1016/j.cyto.2004.11.002

[B12] Mazurek T Zhang L Zalewski A Mannion JD Diehl JT Arafat H Sarov-Blat L O'Brien S Keiper EA Johnson AG Martin J Goldstein BJ Shi Y Human epicardial adipose tissue is a source of inflammatory mediators Circulation 2003 108 2460 2466 10.1161/01.CIR.0000099542.57313.C5 14581396

[B13] Marchington JM Mattacks CA Pond CM Adipose tissue in the mammalian heart and pericardium: structure, foetal development and biochemical properties Comp Biochem Physiol B 1989 94 225 232 10.1016/0305-0491(89)90337-4 2591189

[B14] Marchington JM Pond CM Site-specific properties of pericardial and epicardial adipose tissue: the effects of insulin and high-fat feeding on lipogenesis and the incorporation of fatty acids in vitro Int J Obes 1990 14 1013 1022 2086494

[B15] Manzella D Grella R Marfella R Giugliano D Paolisso G Elevated post-prandial free fatty acids are associated with cardiac sympathetic overactivity in Type II diabetic patients Diabetologia 2002 45 1737 1738 10.1007/s00125-002-0965-8 12552366

[B16] Paolisso G Gualdiero P Manzella D Rizzo MR Tagliamonte MR Gambardella A Verza M Gentile S Varricchio M D'Onofrio F Association of fasting plasma free fatty acid concentration and frequency of ventricular premature complexes in nonischemic non-insulin-dependent diabetic patients Am J Cardiol 1997 80 932 937 10.1016/S0002-9149(97)00548-1 9382011

[B17] Iacobellis G Ribaudo MC Zappaterreno A Iannucci CV Leonetti F Relation between epicardial adipose tissue and left ventricular mass Am J Cardiol 2004 94 1084 1087 10.1016/j.amjcard.2004.06.075 15476634

[B18] Schejbal V [Epicardial fatty tissue of the right ventricle--morphology, morphometry and functional significance] Pneumologie 1989 43 490 499 2813303

[B19] Shibata R Sato K Pimentel DR Takemura Y Kihara S Ohashi K Funahashi T Ouchi N Walsh K Adiponectin protects against myocardial ischemia-reperfusion injury through AMPK- and COX-2-dependent mechanisms Nat Med 2005 11 1096 1103 10.1038/nm1295 16155579 PMC2828682

